# A Novel Laser Vaccine Adjuvant Increases the Motility of Antigen Presenting Cells

**DOI:** 10.1371/journal.pone.0013776

**Published:** 2010-10-29

**Authors:** Xinyuan Chen, Pilhan Kim, Bill Farinelli, Apostolos Doukas, Seok-Hyun Yun, Jeffrey A. Gelfand, Richard R. Anderson, Mei X. Wu

**Affiliations:** 1 Wellman Center for Photomedicine, Department of Dermatology, Massachusetts General Hospital (MGH), Harvard Medical School (HMS), Boston, Massachusetts, United States of America; 2 Department of Medicine, Massachusetts General Hospital (MGH), Harvard Medical School (HMS), Boston, Massachusetts, United States of America; 3 Harvard-MIT Division of Health Sciences and Technology, Boston, Massachusetts, United States of America; New York University, United States of America

## Abstract

**Background:**

Development of a potent vaccine adjuvant without introduction of any side effects remains an unmet challenge in the field of the vaccine research.

**Methodology/Principal Findings:**

We found that laser at a specific setting increased the motility of antigen presenting cells (APCs) and immune responses, with few local or systemic side effects. This laser vaccine adjuvant (LVA) effect was induced by brief illumination of a small area of the skin or muscle with a nondestructive, 532 nm green laser prior to intradermal (*i.d.*) or intramuscular (*i.m.*) administration of vaccines at the site of laser illumination. The pre-illumination accelerated the motility of APCs as shown by intravital confocal microscopy, leading to sufficient antigen (Ag)-uptake at the site of vaccine injection and transportation of the Ag-captured APCs to the draining lymph nodes. As a result, the number of Ag^+^ dendritic cells (DCs) in draining lymph nodes was significantly higher in both the 1° and 2° draining lymph nodes in the presence than in the absence of LVA. Laser-mediated increases in the motility and lymphatic transportation of APCs augmented significantly humoral immune responses directed against a model vaccine ovalbumin (OVA) or influenza vaccine *i.d.* injected in both primary and booster vaccinations as compared to the vaccine itself. Strikingly, when the laser was delivered by a hair-like diffusing optical fiber into muscle, laser illumination greatly boosted not only humoral but also cell-mediated immune responses provoked by *i.m.* immunization with OVA relative to OVA alone.

**Conclusion/Significance:**

The results demonstrate the ability of this safe LVA to augment both humoral and cell-mediated immune responses. In comparison with all current vaccine adjuvants that are either chemical compounds or biological agents, LVA is novel in both its form and mechanism; it is risk-free and has distinct advantages over traditional vaccine adjuvants.

## Introduction

Vaccine adjuvants are traditionally defined as chemical compounds or macromolecules that augment immune responses of co-administered antigen (Ag) with minimal toxicity or long lasting immunity on their own. These agents target innate immune responses through two major mechanisms. Adjuvants like aluminum salts, oil-in-water emulsions, and liposomes facilitate Ag depot and thus Ag-uptake by antigen presenting cells (APCs). Other adjuvants like monophosphoryl lipid A (MPL), CpG, or poly I:C, activate APCs by binding to Toll-like receptors [1]. All these adjuvants cause inflammation at the site of injection and with local reactogenicity, and many of them also have a potential for long term side effects and therefore have not been approved for human use. The most widely used adjuvant in the clinics has been aluminum-based mineral salts (alum) over the past 80 years [2].

Alum has a good track record of safety and has been widely and successfully used in many licensed vaccines [3]. However, some limitations of alum have been described. For instance, it is a rather weak adjuvant when used with certain types of vaccines like influenza hemaggulutinin (HA) antigens [4], typhoid vaccine [5], Haemophilus influenzae type b (Hib) capsular polysaccharide conjugated to tetanus toxoid [6], or recombinant protective antigen of anthrax after freezing [7]. Alum appears to be potent in primary immunizations, but has a limited ability to boost humoral immune responses in second and third doses [8]. It also affects little in cell-mediated immunity and induces primarily Th2 immune responses [3]. Moreover, while alum adjuvant causes some low level local reactogenicity in most of vaccinees, it has been linked to the macrophagic myofasciitis (MMF) in some people who received intramuscular alum-adjuvanted vaccines in France [9]. In addition to side effects, alum is a non-crystalline gel and antigen must be adsorbed onto the highly charged aluminum particles for the adjuvant to be potent. At least two serious effects result from use of alum. First, freezing, lyophilization or cold storage that results in separation of antigen from the aluminum particles would cause a loss of the adjuvant potency [10]–[12]. Secondly, the biophysical structure and stability of the resultant product are difficult to assay as an alum complex. Therefore, alternative vaccine adjuvants that are non-toxic, consistently effective, and easily used have been hunted for in the past thirty years.

Lasers have been applied to medicine for decades, and their therapeutic potential is still being actively explored. Conventional medical applications of lasers with a high power have largely been designed to destroy unwanted tissues, such as laser surgery, laser ablation or thermotherapy. Photodynamic therapy kills cancer cells via photoreactive compounds [13], which stimulates long-term, systemic tumor immunity and eradicate distal tumors in some cases [14]. Low-power lasers are used to seal blood vessels or “weld” tissues together, to accelerate the healing of wounds and burns [15]; [16] or to modulate immune responses as adjuvant therapy [17]. Research on the use of laser to boost immunization has been very limited. Femtosecond laser was shown to sufficiently enhance DNA delivery into cells and induce immune responses to the encoded antigen [18]. Onikienko and colleagues reported in a Russian journal that pre-illumination of skin with a pulsed copper vapor laser at a high power (0.6 W) enhanced humoral immune responses provoked by vaccines *i.d.* injected at the site of laser exposure in a manner dependent on heat shock protein (HSP)-70 and inflammatory cytokines [28].

The current investigation aims at the development of a practical, safe laser vaccine adjuvant (LVA) capable of boosting both humoral and cell-mediated immune responses against protein-based vaccines, with few side effects. We show here that brief laser illumination at a specific laser setting increases the mobility of APCs and thus an efficacy of antigen capture by the cells, without incurring any inflammation or reactogenicity. Laser-mediated increases in antigen capture by APCs greatly strengthened humoral and cell-mediated immune responses against either a model or a clinical vaccine. LVA represents a novel, potent vaccine adjuvant with few side effects.

## Materials and Methods

### Mice

Male BALB/c mice at 6–8 weeks of age were purchased from Charles River Laboratories (Wilmington, MA). MHC II-EGFP mice expressing MHC class II molecule infused into enhanced green fluorescent protein were a kindly gift of Drs. Boes and Ploegh [19]. All mice were housed in conventional cages in the animal facilities of Massachusetts General Hospital (MGH) in compliance with institutional guidelines. The study was reviewed and approved by the MGH Subcommittee of Research Animal Studies.

### Devices

A Q-switched 532-nm Nd:YAG laser with a pulse width 5–7 ns, beam diameter 7 mm, and frequency 10 Hz was used in the study unless otherwise indicated (Spectra-Physics Inc., Mountain View, CA). Average output powers were measured by a power meter (Ophir Optics, Inc., MA) prior laser illumination. Skin temperature (Tm) was monitored during laser illumination by an infrared camera focusing on the center of the illumination area (FLIR Systems, Boston, MA).

### Immunizations

Mice were anesthetized by intraperitoneal (*i.p.*) injection of a mixture of ketamine (80 mg/kg) and xylazine (20 mg/kg) and the lower dorsal hair of the mice was removed by shaving and a hair removal lotion (Church&Dwight Co.). The skin was exposed next day to laser for 2 min at 0.3 W unless otherwise indicated after the mice were anesthetized similarly. The mice were then immunized by intradermal (*i.d.*) administration into the laser-illuminated site with either an indicated dose of ovalbumin (OVA, Grade V, Sigma, St. Louis, MO) or 2009–2010 seasonal influenza (flu) virus vaccine (Fluvirin, Novartis). Mice in control groups were treated and immunized similarly except for no laser illumination. In some experiments, OVA solution was passed through a Detoxi-Gel column to remove contaminated endotoxin according to the manufacturer's instruction (Thermo Scientific, Rockford, IL). Effects of laser on a booster vaccination were evaluated with flu vaccine by vaccination at the contralateral site following laser illumination at an interval of 3 weeks. To determine the effect of skin Tm on laser-mediated immune enhancement, skin Tm was maintained at 42°C by a 10×10×100 mm^3^ steel bar that was vertically immersed in a 44°C water bath with its top 1 cm above the water surface. After warming the skin for 2 min, the mice were immunized at the warm skin with OVA as above. In a separate series of experiments aimed at addressing laser adjuvant effects on intramuscular (*i.m.*) vaccination, a hair-like optical fiber with a 5-mm diffusing tip coated with ZnO-Epoxy resin was inserted perpendicularly into the posterior thigh muscle and lighted up by a long-pulsed laser (KTP/532 nm, Aura; Laserscope, San Jose, CA) at 0.3 W for 1 minute followed by *i.m.* vaccination of 50 µg OVA.

### Blood collection and serum antibody titer detection

Blood samples of ∼30 µl were collected by nicking the tail vein before or at the indicated times after immunization. Serum was prepared by centrifugation to remove the cells and kept at −80°C till analysis. Serum antigen specific IgG levels were detected by ELISA (Enzyme-linked immunosorbent assay) with 100 µg/ml OVA or 1 µg/ml Flu vaccine as a coating antigen (Ag). Antibody (Ab) titer was determined by the dilution factor with an OD_490 nm_ absorbance ≥0.2. No mice receiving laser illumination only or before immunization had detectable levels of antibody directed against OVA or flu vaccine during the course of this study.

### Intravital confocal imaging

The posterior thigh skin of MHC II-EGFP transgenic mice was either left untreated or exposed to laser for 2 min at 0.3 W, followed by *i.d*. injection of endotoxin-free OVA or PBS as controls. Dermal GFP^+^ cells were imaged 5 hrs later by intravital confocal microscopy, during which body Tm was maintained at 36°C. Every twelve overlapping images of dermal GFP^+^ cells at the site of immunization or laser illumination were acquired and merged into single large-field images using Photoshop CS3.0 software. To analyze the migratory ability of APCs, the time lapse images of dermal GFP^+^ cells at a specific area were acquired every 30 seconds for 20 minutes and pseudopods of about 30 randomly selected cells were tracked individually by Image J software.

### Detection of Ag-captured dendritic cells (DCs)

To determine the number of Ag-captured DCs in the skin, fluorescently labeled OVA (Alexa Fluor 647-conjugated OVA or AF647-OVA) was *i.d*. injected into the laser-exposed skin as above. Full thickness of the skin about 7×7 mm^2^ at the site of Ag-injection was excised 6 or 24 hrs later, washed thoroughly, cut into small pieces, and digested in 0.2% collagenase D (Roche Diagnostics, Mannheim, Germany) supplemented with 0.6 U/ml dispase (Gibco, Invitrogen, Carlsbad, CA) and 2% fetal bovine serum (FBS) in PBS at 37°C for 2 hr with intermittent vortexing. Single cell suspensions were prepared by passing the digest through a 40-µm cell strainer and stained with anti-CD11c antibody (N418) (eBioscience, San Diego, CA), a marker for DCs. The number and percentage of CD11c^+^AF647-OVA^+^ cells on gated CD11c^+^ cells were acquired on FACSAria equipped with FACSDiva software (Becton Dickinson) and analyzed using FlowJo software. To evaluate the number of Ag-captured DCs in the lymph node (LN), LNs were dissected and minced against a 40-µm cell strainer. The number and percentage of CD11c^+^ AF647-OVA^+^ cells were analyzed as above.

### Measurement of cell-mediated immunity

CD4+ T cell-mediated immunity was evaluated in mice three weeks after primary immunization with OVA at the laser-exposed site or OVA alone. Ag-specific T cells were activated by *i.p.* injection of 40 µg OVA and expanded *in vivo* for one week. Splenocytes were isolated and stimulated with 50 ng/ml PMA and 750 ng/ml ionomycin in the presence of 0.1% Golgi-Plug for 5 hrs at 37°C with 5% CO_2_. Cells were then stained with PE-anti-CD4 (GK1.5), fixed, and permeabilized in a permeabilization buffer per the manufacturer's instruction, followed by staining with Alexa Fluor 647-anti-IL4 (11B11) and FITC-anti-IFNγ (XMG1.2). Alternatively, cell-mediated immunity against OVA, including both CD4^+^ and CD8^+^ T cells, was assessed three weeks after primary immunization by *in vitro* stimulation assays. Briefly, popliteal draining LNs were isolated and total cells were counted and stimulated overnight with 10 µg/ml OVA, 4 µg/ml anti-CD28 monoclonal antibody 37.51 (BD Bioscience Pharmingen, San Diego, CA). The stimulation continued for another 6 hrs after addition of 0.1% Golgi-Plug to the culture. The stimulated cells were stained with FITC-anti-CD4 (GK1.5) or PerCP-Cy5.5-anti-CD8 (53-6.7), fixed, and permeabilized, followed by staining with anti-IL4 and anti-IFNγ as above. The numbers of CD4^+^IL4^+^ and CD4^+^ IFNγ^+^ cells or CD8^+^IL4^+^ and CD8^+^IFNγ^+^ cells were analyzed on FACSAria and expressed as absolute numbers of CD4^+^IL4^+^ and CD4^+^IFNγ^+^ cells or CD8^+^IL4^+^ and CD8^+^IFNγ^+^ per LN. All the reagents were purchased from Biolegend, San Diego, CA unless otherwise indicated.

### Histological examination

The lower dorsal skin of mice was exposed to laser for 2 min at 0.3 W or *i.d.* injected with 10 µl alum adjuvant (Imject Alum, Thermo Scientific, Rockford, IL). Full thickness of the skin at the site of laser illumination or alum injection was excised after 2 hrs or 1 or 3 days, fixed in 10% formalin and subjected to a standard histological examination.

### Quantitative real-time PCR

To analyze inflammatory cytokine gene expression following laser illumination, the full thickness of the skin area about 7×7 mm^2^ was excised 6 hrs after laser illumination for 2 min at 0.3 W or alum injection as above. Total RNA was extracted, reverse-transcribed, and amplified by real-time PCR using a SYBR Green PCR kit (Applied Biosystems, Foster City, CA) on an Mx4000™ Multiplex Quantitative PCR System (Stratagene). Threshold cycle (Ct) was used to calculate the relative template quantity as the manufacturer's recommendation using β-actin as an internal control. The basic gene expression level was set at 1 when analyzing the data. The primers used were: forward, CCCTCACACTCAGATCATCTTCT and reverse, GCTACGACGTGGGCTACAG for TNFα; forward, GCAACTGTTCCTGAACTCAACT and reverse, ATCTTTTGGGGTCCGTCAACT for IL-1β; forward, TAGTCCTTCCTACCCCAATTTCC and reverse, TTGGTCCTTAGCCACTCCTTC for IL-6; forward, TTAAAAACCTGGATCGGAACCAA and reverse, GCATTAGCTTCAGATTTACGGGT for CCL2; and forward, GGCTGTATTCCCCTCCATCG and reverse, CCAGTTGGTAACAATGCCATGT for β-actin.

### Statistical analysis

Student *t*-test or one-way ANOVA was used to analyze the difference between groups or among multiple groups, respectively. P values were calculated by PRISM software (GraphPad, San Diego, CA).

## Results

### Determination of a safe laser setting

**Figure 1:**
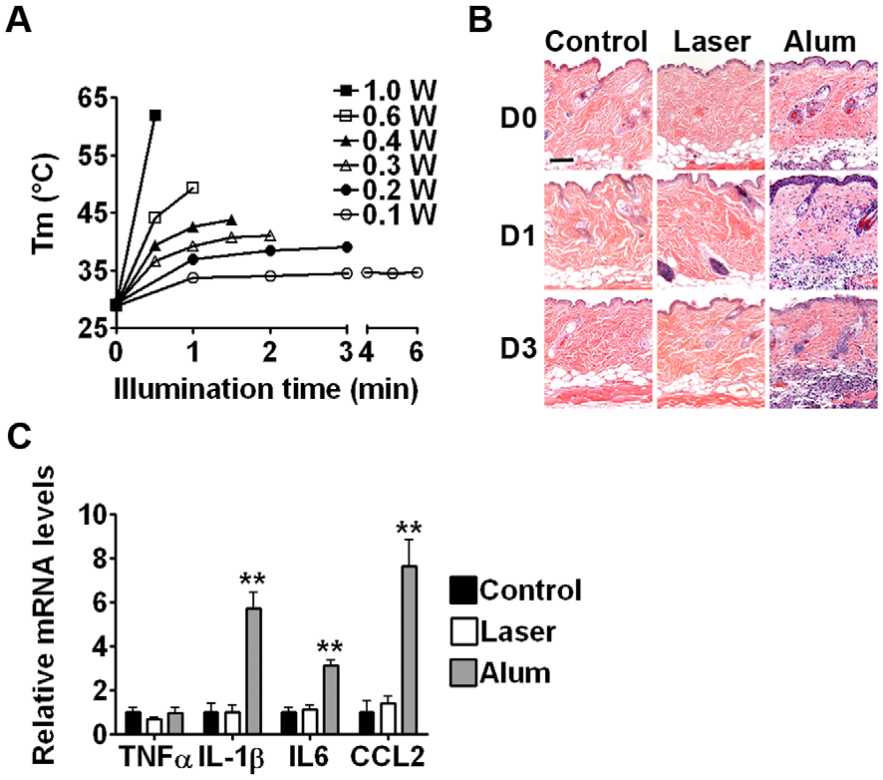
No significant alteration in skin histology after laser illumination for 2 min at 0.3 W. The lower dorsal skin of mice was exposed to laser at indicated output powers and times. Skin Tm was monitored during the exposure by an infrared camera (**A**). Histological examination was performed after 2 hrs (D0), 1 day (D1) or 3 days (D3) in the skin that was either illuminated with laser for 2 min at 0.3 W or *i.d.* injected with alum adjuvant (10 µl) or PBS as controls (**B**). Full thickness of skin tissues treated for 6 hrs as in **B** was evaluated for inflammatory cytokine expression by real-time RT-PCR (**C**). Scale bar in **B**, 100 µm. Data are representative of at least three experiments with similar results in **A** and **B**. Data in **C** are the means ± standard errors of the mean (SEM) of six samples. **, p<0.01.

To define a laser setting that could boost immune responses without incurring any tissue damage, skin temperature was monitored by an infrared camera during light illumination, as the photothermal reaction is considered to be the primary cause for laser-induced tissue damage. Skin temperature rose with increasing output powers of laser and times of illumination. Average output powers of 0.6 W or higher damaged skin instantly, evidenced by skin whitening and shrinkage, concurrent with elevation of skin temperature to 50°C or 60°C, respectively, in less than a minute (Figure 1A). On the contrary, an output power of 0.3 W or lower did not raise the skin temperature higher than 41°C or cause discoloration of the skin visibly even for an extended period of illumination (Figure 1A). Laser illumination at 0.3 W for 2 min, 90 J/cm^2^ also caused little alteration in tissue histology at the site of illumination when examined on day 0, 1 or 3 post illumination (Figure 1B). In accordance to this, we observed, on a high resolution, no overt cell death or leukocyte infiltration in these tissue samples. In contrast, an output power of 0.4 W or higher damaged tissues with apparent cell death in the epidermis and was thus precluded from our studies. In parallel studies, intradermal administration of alum adjuvant stimulated vigorous infiltration of leukocytes into both dermal and subcutaneous tissues as early as 2 hrs (day 0), which was persistent for weeks, concurrent with the production of high levels of inflammatory cytokines such as IL-1β, IL6, and CCL2 (Figure 1B and C). No increases in the expression of inflammatory cytokines including TNFα, IL-1β, IL6, and CCL2, were observed by laser illumination when compared to controls (Figure 1C).

### Laser enhances the motility of APCs

**Figure 2:**
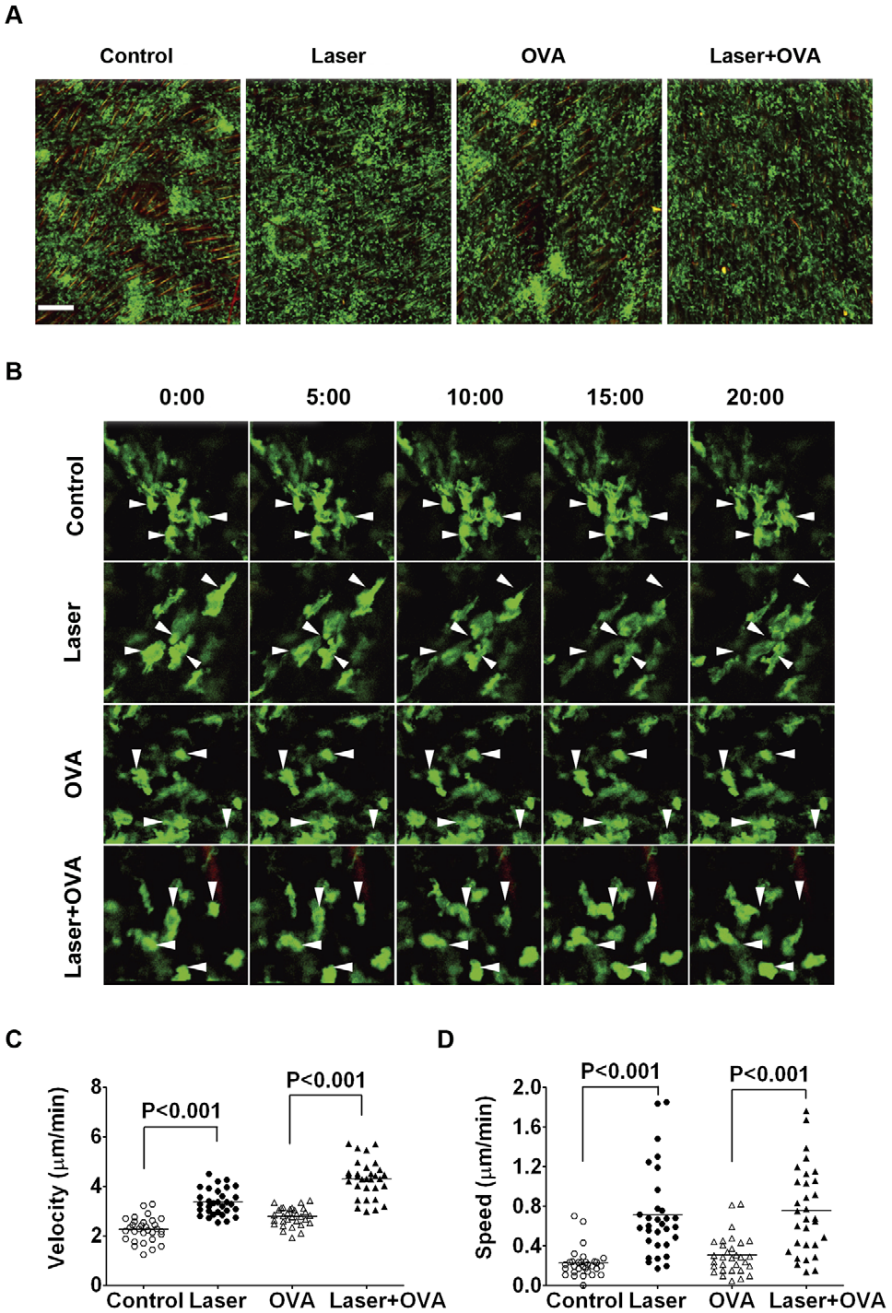
Laser increases the mobility of dermal APCs. **A**. Altered distribution of dermal APCs after laser illumination. Distribution of dermal APCs in MHC-II EGFP transgenic mice was analyzed by intravital confocal microscopy after 5 hrs of laser illumination for 2 min at 0.3 W with or without OVA administration. Scale bar: 200 µm. **B**. Increased motility of individual cells after laser illumination. Representative time-lapse images demonstrated migratory behaviors of dermal APCs within a 20 minute period of recording: arrows indicate the original location of the cell. **C** and **D**. Quantification of cell motility affected by laser illumination. Mean mobile velocity (**C**) or migratory speed (**D**) of randomly selected pseudopods was imaged in 5 hrs after laser illumination and OVA injection and analyzed by Image J software. Each symbol represents data of a single pseudopod. *n* = 31.

APCs are the primary targets for most of adjuvants [1]. We therefore addressed whether this non-inflammatory, safe laser could affect the activity of skin APCs. To this end, APCs in MHC II-EGFP transgenic mice were tracked by intravital confocal microscopy after 5 hrs of laser illumination for 2 min at 0.3 W [19]; [20]. Intravital imaging of the dermal layer, where a majority of antigen was taken up by APCs following *i.d.* administration, revealed that a majority of APCs formed discernible cell islands or clusters in the control (Figure 2A). These cell clusters appeared to be dispersed into single cells spreading evenly following laser illumination (Figure 2A). While OVA injection also provoked cell spreading, the degree of dispersion seemed much less prominent as compared to that seen with laser illumination. A combination of laser and OVA further dispersed the cells, as reflected by a drastic increase in the number of single cells in the dermis, concomitant with a great decrease in the number of cell clusters over OVA alone (Figure 2A).

To address whether laser-induced redistribution of APCs was ascribed to increased cell motility, we tracked the behavior of APCs in the dermal layer 5 hrs after laser illumination by acquiring the time-lapse images of individual cells every 30 seconds for total 20 minutes with intravital confocal microscopy. Dermal GFP^+^ cells, mostly DCs and macrophages, were constantly changing their cell shapes, albeit slowly, and extending pseudopods for environmental surveillance, but most of them remained at their original locations during a 20 min period of recording in the control (Figure 2B, 1^st^ panel). On the contrary, the cells in the laser-treated mice showed a high migratory ability, moving away from their original locations during the same period of time, as indicated by an increased distance between arrows and the individual cells over times (Figure 2B, 2^nd^ and 4^th^ panels). OVA injection also increased migration of APCs, albeit to a lesser extent (Figure 2B, 3^rd^ panel). Strikingly, when OVA was administrated into the site of laser illumination, a synergistic effect was observed on APC motility. Quantification of the cell motility by tracking of randomly selected 30 cells revealed that laser illumination alone increased pseudopod mobile velocity, a rate of change in their position regardless of direction over to time, from 2.26±0.09 µm/min in the control, to 3.35±0.09 µm/min in the laser-treated skin (Figure 2C, p<0.001). When laser treatment was combined with OVA immunization, the pseudopod mobile velocity was accelerated to 4.29±0.13 µm/min, which was significantly higher than 2.78±0.07 µm/min observed with OVA alone (p<0.001). Likewise, the cell pseudopod migratory speed as measured by a traveling distance per min was faster with laser illumination irrespective of whether or not OVA was administered (0.71±0.08 vs. 0.23±0.03 µm/min in the absence of OVA and 0.76±0.08 vs. 0.31±0.03 µm/min in the presence of OVA, p<0.001) (Figure 2D). Consequently, over 64% of APC pseudopods in the laser-treated or laser+OVA-treated groups had a migratory speed above 0.5 µm/min, whereas less than 10% of pseudopods in control and OVA alone groups showed a migratory speed above 0.5 µm/min (Figure 2D). Similar increases in the motility of APCs were observed in 0.5 or 16 hr, but not at 24 hr, after laser illumination, which probably occurred upon laser illumination (data not shown).

### Laser illumination enhances Ag-uptake by skin DCs

**Figure 3:**
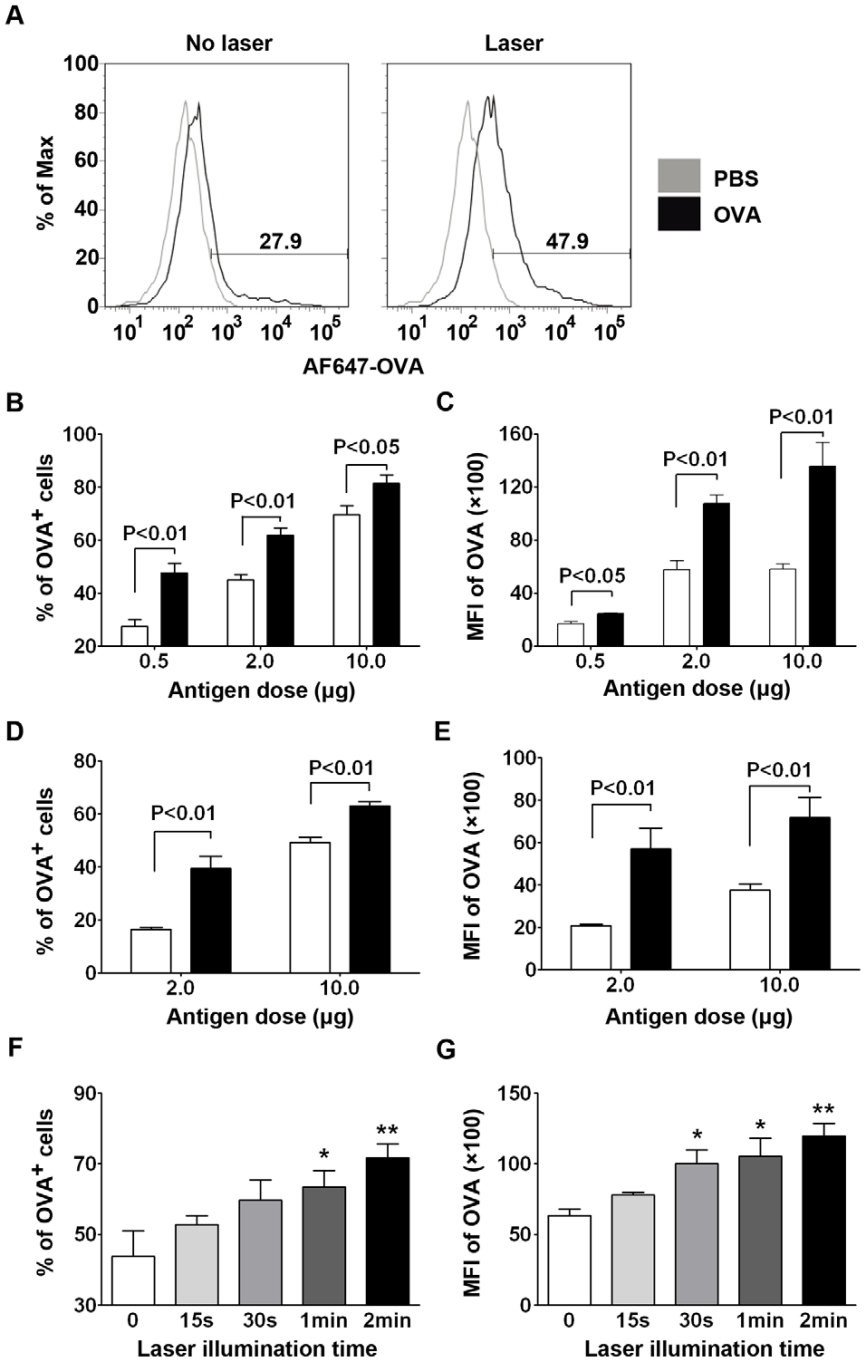
Laser enhances Ag-uptake by DCs in the skin. **A.** Representative flow cytometry histograms showing percentages of OVA^+^ CD11c^+^ cells. Single cell suspension was prepared from full thickness of the skin 6 hrs after 0.5 µg AF647-OVA in 20 µl or 20 µl PBS was *i.d.* administrated into the site of laser illumination or control. The cells were stained with anti-CD11c antibody, and analyzed for percentages of OVA^+^CD11c^+^ cells on the gated CD11c^+^ cell population. Percentages of OVA^+^ CD11c^+^ cells (**B** and **D**) and mean fluorescence intensity (MFI) of AF647-OVA (**C** and **E**) were analyzed at 6 hr (**B** and **C**) or 24 hr (**D** and **E**) after *i.d.* administration of AF647-OVA at the indicated doses. *n* = 6, blank bar, AF647-OVA alone; and black bar, laser + AF647-OVA. **F** and **G**. A light dose-dependent increase in Ag-uptake by DCs. The lower dorsal skin of mice was illuminated with laser at 0.3 W for the indicated time, corresponding to a light dose of 11.3, 22.5, 45, or 90 J/cm^2^, respectively. AF674-OVA at 2 µg/ mouse was injected into the laser-illuminated site and 6 hrs later, percentages of OVA^+^ CD11c^+^ cells (**F**) and MFI of AF647-OVA (**G**) were analyzed and expressed as above.* and **, p<0.05 and 0.01, respectively.

A significant increase in the motility of APCs resulting in a high number of single APCs in the dermis was expected to augment Ag-uptake at the site of Ag injection. To address this, 0.5 µg fluorescently labeled OVA (AF647-OVA) was *i.d.* administered into laser-exposed skin, followed by flow cytometric analysis of CD11c^+^AF647-OVA^+^ cells at the Ag-injection site (Figure 3A). While percentages of Ag^+^DCs were increased proportionally with the amounts of OVA injected, significantly higher levels of Ag^+^DCs were consistently attained at 6 hrs after laser illumination and/or Ag injection, in the laser-treated group than in non-treated group at all antigen concentrations tested (Figure 3A&B). At the lowest Ag concentration, approximately 50% of DCs had taken up the Ag at laser-treated site as compared to 27% of Ag^+^DCs in non-illuminated control (Figure 3A&B). A similar increase in the percentage of OVA^+^ CD11c^+^ cells was also observed in 24 hrs (Figure 3D). Not only did a high proportion of DCs capture the Ag but also more Ag was taken up by individual DCs owing to laser pre-illumination, manifested by a significantly higher level of OVA mean fluorescent intensity (MFI) in individual cells at all Ag concentrations tested (Figure 3C&E). The Ag-uptake activity of DCs was light-dose dependent (Figure 3F&G): the longer illumination was and the higher Ag-uptake activity resulted. Notably, percentages of Ag^+^DCs and MFI of AF647-OVA were reduced at 24 hr as compared to 6 hr in both laser-treated and control groups, presumably due to DC emigration as well as antigen processing during this period of time. Despite an increase in the number of OVA^+^CD11c^+^ cells, we did not observe a significant difference in the percentage of CD11c^+^ cells with or without laser treatment (data not shown), suggesting that DC influx is unlikely to contribute to the increased number of OVA^+^CD11c^+^ cells, in agreement with a highly precise nature of laser.

### Laser illumination enhances Ag-uptake in draining lymph nodes

**Figure 4:**
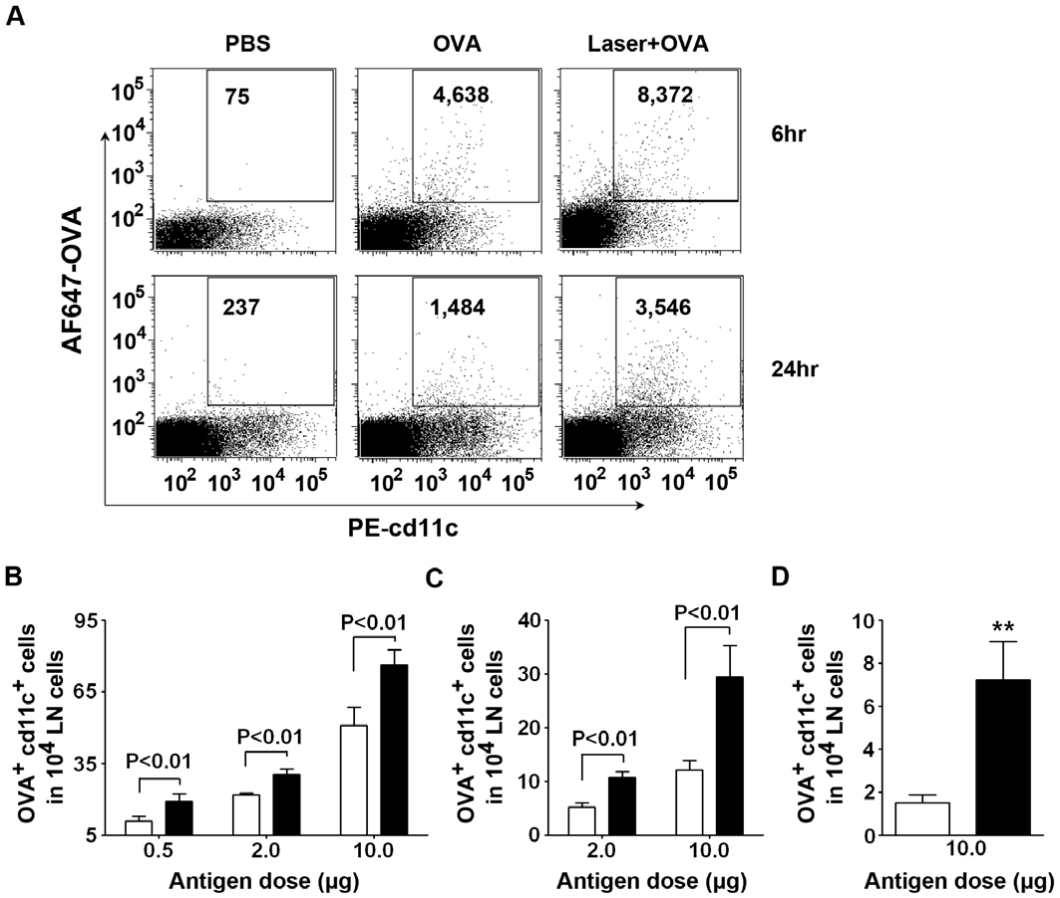
Laser increases Ag-uptake by DCs in the draining lymph node. **A**. Representative flow cytometry profiles showing the number of OVA^+^CD11c^+^ cells. Single cell suspensions were prepared from the draining LN (inguinal) after 6 or 24 hrs of laser illumination and *i.d.* injection of 10 µg AF647-OVA. The cells were stained with anti-CD11c antibody and analyzed as Figure 3. The absolute numbers of CD11c^+^OVA^+^ cells were given in the profile in one million cells counted. **B** and **C**. An increase in the number of Ag-captured DCs in the 1° draining LNs. The cells were prepared from the inguinal LN at 6 hr (**B**) or 24 hr (**C**) after laser illumination and AF647-OVA injection at the indicated dose and analyzed as above. Data are means ± SEM of absolute numbers of CD11c^+^OVA^+^ cells in 10^4^ LN cells from draining lymph nodes analyzed as **A**. *n* = 6; blank bar, AF647-OVA alone and black bar, laser + AF647-OVA. **D**. An increase in the number of Ag-captured DCs in the 2° draining LN at 24 hrs after 10 µg AF647-OVA was *i.d.* administered. The cells isolated from the ipsilateral axillary LN were analyzed and the data were expressed as above.

Consistent with an increase in the motility and Ag-uptake of DCs in the skin after laser illumination, the number of Ag^+^DCs in the draining lymph nodes (LNs) was also significantly greater at 6 or 24 hr after varying concentrations of OVA were administered into the site of laser illumination than into a non-illuminated control site (p<0.01, Figure 4). Strikingly, an almost 5-fold increase in the number of OVA^+^ DCs was observed in the secondary axillary draining LN in LVA-treated group as compared to non-LVA controls (Figure 4D), presumably due to either the enhanced free antigen flow and/or the enhanced migration of Ag+DCs to the 2° draining LN by laser illumination (Figure 4B vs 4C). Only a few basal Ag-uptake DCs were seen in contralateral or mesenteric LNs, which were not affected by laser illumination (data not shown).

### Laser augments and prolongs humoral immune responses

**Figure 5:**
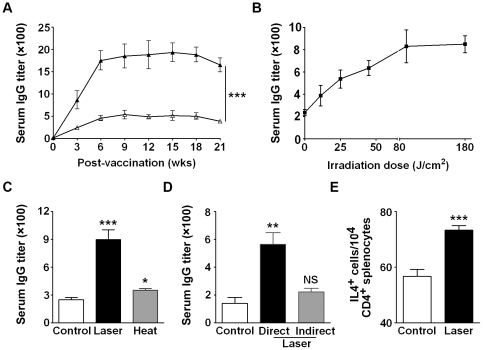
Laser enhances humoral immune response induced by OVA. **A**. Laser significantly augments and prolongs the production of serum OVA-specific antibody. Serum OVA-specific antibody was detected at indicated times after *i.d.* injection of 40 µg OVA with (filled) or without laser illumination (blank). **B**. A light-dose dependent augmentation of OVA-specific antibody production. The lower dorsal skin of mice received an increasing laser dose from 11.3 to 22.5, 45, 90, or 180 J/cm^2^, which corresponded to irradiation at 0.3 W for 0.25, 0.5, 1, 2, and 4 min, respectively. **C**. Immune-enhancement effect of laser cannot be recapitulated with a Tm rise in the skin. Heat: the skin was warmed with a 42°C metal rod for 2 min mimicking the Tm rise caused by laser illumination as described in Materials and Methods. **D**. Laser-mediated immune enhancement is area-restricted. A significant increase in OVA-specific antibody production was attained only when OVA was injected into the illuminated site (direct) but not in a distal site (indirect). Serum Ab titers were measured 3 wks after immunization in **B**∼**D**. **E**. Laser increases the number of CD4^+^IL4-secreting T cells. T cells of OVA-immunized mice were activated *in vivo* for one week by *i.p.* injection of 40 µg OVA. CD4^+^IL4-secreting cells were analyzed by flow cytometry after surface staining with anti-CD4 antibody and then intracellular staining with anti-IL-4 antibody. Data are expressed as means ± SEM of absolute numbers of CD4^+^IL4^+^ cells in 10^4^ CD4^+^ splenocytes. *n* = 6 for each group except for **B** in which 3 mice were used in each time point. *, **, ***, *p*<0.05, 0.01, and 0.001, respectively, using one-way analysis of variance (ANOVA) in **C** and **D**, and student *t*-test in **A** and **E**.

Immune effects of this safe laser were next evaluated using a model antigen OVA. Laser illumination at 0.3 W for 2 min with a dose of 90 J/cm^2^ boosted OVA-specific antibody (Ab) production by 300∼500% over intradermal OVA injection alone (Figure 5A, p<0.001). The high titer of antibody production was sustained for more than 15 weeks following a single immunization (Figure 5A). Production of OVA-specific Ab was proportionally elevated with an increasing laser dose up to 90 J/cm^2^, reaching a plateau between 90∼180 J/cm^2^ (Figure 5B), in agreement with the light dose-dependent Ag-uptake activity of dermal DCs (Figure 3F). Similar laser adjuvant effects were also observed when endotoxin-removed OVA was used for immunization (data not shown), ruling out that the immune-enhancing effect was attributed in part to endotoxin contamination. Cell-mediated immunity against OVA was also assessed by analysis of CD4^+^IL4-secreting splenocytes in these mice. Mice receiving laser plus OVA produced a significantly higher number of CD4^+^IL4-expressing cells than mice immunized with OVA alone (Figure 5E, p<0.001). But, no such an increase was observed in IFN-γ-secreting CD4^+^ or CD8^+^ T cells with this low-power laser illumination in the animals regardless of whether the specific T cells were stimulated and expanded *in vivo* or *in vitro* cell culture (data not shown). However, Th1 immune responses were observed when an increase in a light dose >180 J/cm or an output power >0.3 W was applied prior to *i.d*. administration of OVA, concomitant with some tissue damage that was self-resolved in two or three days (data not shown).

We noted that although laser illumination elevated skin temperature, laser-mediated immune enhancement was not solely ascribed to the photothermal effect. As shown in Figure 5C, when the skin area of 1 cm^2^, a size that is larger than that of the laser beam (0.4 cm^2^), was warmed to 42°C with a metal bar for 2 min followed by antigen injection as above, OVA-specific Ab production was increased only by 20%. Moreover, if antigen was injected into a distal site (indirect), for instance, 1 cm away from the laser-illuminated site, the immune-enhancing potential decreased substantially (Figure 5D). Thus, Ag delivery directly into the site of laser illumination is a key.

### Laser augments not only primary but also booster immune responses against flu vaccine

**Figure 6:**
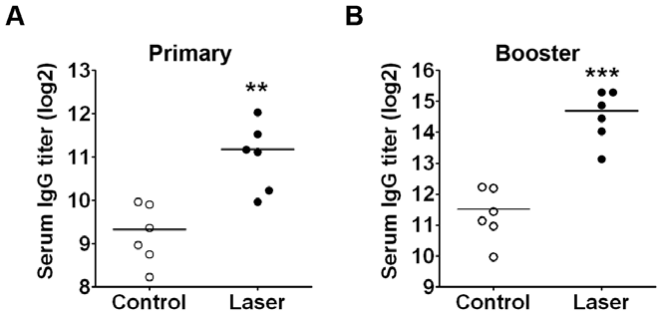
Laser enhances flu vaccine-induced immune responses in both primary and booster immunizations. Laser augments not only primary (**A**) but also booster (**B**) immune responses. Flu-specific antibody was measured by ELISA at 3 weeks after the primary immunization with 0.3 µg flu vaccine at the site of laser illumination or control as Figure 5 (**A**). A booster immunization was carried out at the contralateral side similarly three weeks later after blood collection for assessing primary immune responses. Flu-specific antibody was detected in two weeks as above (**B**). Each symbol represents the data from individual mice. **, P<0.01; and ***, P<0.001.

To test immune-enhancing effects of LVA on a clinically approved vaccine, the newest season flu vaccine (2009–2010) was evaluated for both primary and booster immunizations. Laser pre-illumination enhanced the production of flu-specific antibody by 400% in primary vaccination (Fig. 6A, p<0.01) and by 900% in a booster immunization compared to vaccine control (Fig. 6B, p<0.001). The synergistic enhancement of specific antibody production in the second immunization over the first one is pivotal for flu vaccines as most people receive flu vaccines annually and are considered being primed for flu vaccines due to cross-reaction among new and previous flu vaccines.

### LVA enhances both humoral and cell-mediated immunity induced by intramuscular immunization

**Figure 7:**
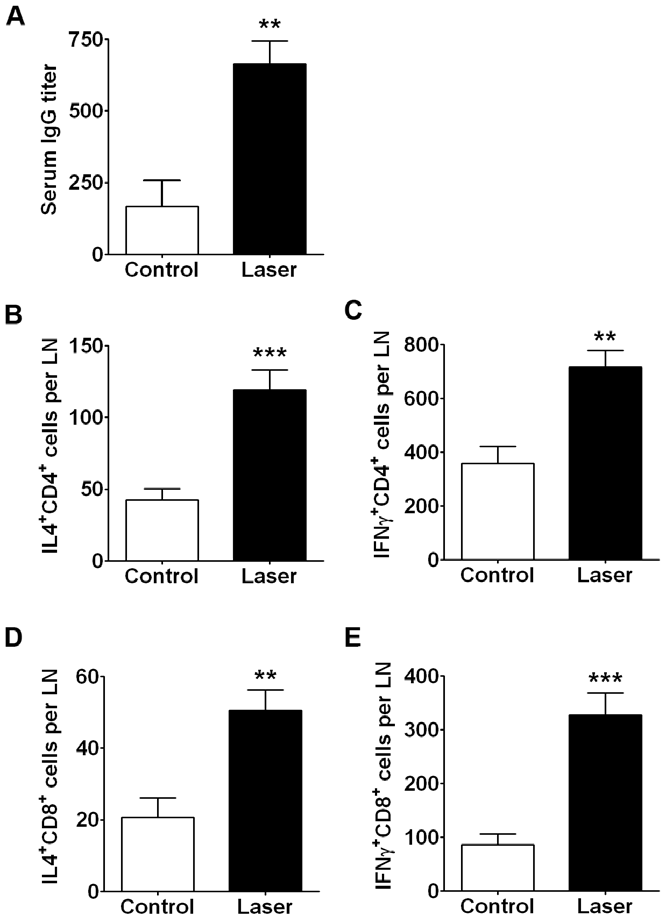
Laser enhances cell-mediated immune responses induced by *i.m.* immunization. The posterior thigh muscle of BALB/c mice was illuminated by a 532-nm laser (KTP/532) delivered by a hair-like diffusing optical fiber followed by *i.m.* vaccination with 50 µg OVA (laser). Control groups received OVA immunization similarly without laser illumination (control). OVA-specific antibody (A) and IL4- and IFNγ-secreting CD4^+^ and CD8^+^ cells in the dLNs were analyzed three weeks later. OVA-specific Ab in the plasma was detected by ELISA as figure 5. The numbers of IL4^+^-secreting CD4^+^ (B) and CD8+ cells (D) and IFNγ^+^-producing CD4^+^ (C) and CD8^+^ (E) cells per LN were identified by flow cytometric analyses after surface staining with anti-CD4 or anti-CD8 antibody followed by intracellular staining with anti-IL4 or anti-IFNγ Ab. n = 6, **, P<0.01; ***, P<0.001.

The majority of current vaccines are intramuscularly administered. In an attempt to explore the universal use of the LVA, we tested whether laser could enhance immunity induced by *i.m.* immunization. To illuminate muscle, a hair-like diffusing optical fiber was made in house by coating ZnO-Epoxy resin at the tip about 5 mm in length, and inserted into the posterior thigh muscle. The optical fiber was then lighted up by 18 ms, 2 Hz at 0.3 W for 1 min equivalent to 45 J/cm^2^ on the skin, after which OVA was *i.m.* administered slowly into the illuminated muscle. Control mice received the same procedure except for not lighting up the optical fiber after its insertion. A significant increase in the production of OVA-specific Ab was attained by laser illumination of muscle over non-treated muscle at a level resembling that seen with *i.d.* vaccination (Figure 7A). In marked contrast, however, an increase in Th1 immune responses was observed following laser illumination of muscle prior to *i.m.* vaccination (figure 7). The numbers of IFNγ-producing CD4^+^ and CD8^+^ T cells were 717 or 328, respectively, in the draining LN, which represent a 100% increase for CD4^+^ cells or 277% for CD8^+^ T cells over Ag alone (Figure 7C and 7E). Laser illumination prior to vaccination also resulted in a greater number of IL4^+^-producing CD4+ and CD8^+^ T cells as compared to Ag alone (Figure 7B and 7D). The results demonstrate the ability of LVA to boost cell-mediated, in particular, CD8+ T cell mediated immune responses.

## Discussion

This investigation provides proof of concept evidence that brief illumination of skin or muscle with non-destructive, safe, green laser can prime the body to a stronger response to a protein prototype vaccine that, by itself, induces relatively weak immune responses. Although augmentation of humoral immune responses by LVA was comparable or might be slightly inferior to alum adjuvant (data not shown), LVA caused little toxicity, local inflammation or reactogenicity, whereas alum adjuvant induced vigorous inflammation, concurrent with skin rash and redness that were persistent for weeks. Foremost, LVA boosts Th1-mediated immune responses that are critical for protection against diseases caused by intracellular pathogens such as viruses, parasites and mycobacterium when applied intramuscularly. In contrast, alum-based vaccine adjuvant has little effect on CD8+ T cell immune responses [3]; [8]. In addition, LVA does not involve administration of any foreign or self substance into the body apart from the antigen itself so that no adjuvant-related complex can be formed with host tissue even after repeated uses, by which a self-destructive immune cross-reaction, also called “molecular mimicry” that can potentially cause long-term side effects, can be effectively prevented. On the contrary, with few exceptions, other adjuvants are foreign to the body and have the potential to cause adverse reactions in the long term if routinely used, in addition to their local inflammation and reactogenicity [21]–[23]. Apart from side effects, LVA is not a chemical or a compound and thus there is no need of a specific formulation procedure to attain a stable mixture between a specific Ag and an adjuvant. Yet, all current vaccine adjuvants require an optimal formulation between vaccine and adjuvant which must be demonstrated to meet pre-determined specifications as physical and biological stability. Meeting these requirements continues to be challenging for some vaccines. For instance, mixing the new circumsporozoite protein antigen RTS with AS04 unexpectedly blocked protective immunity even though RTS in an oil-in-water emulsion containing MPL induced protection against infection of *Plasmodium falciparum* [24]. Thirdly, the laser “adjuvant” can be used immediately and unlimitedly at any time, and does not require cold chain storages. This benefit is of particular significance in preparing for an outbreak of a new strain or flu pandemic such as the 2009 emergence of H1N1 or for biological attacks owing to their unpredictability in both the scale and the timing. Finally, LVA may be effective as a universal co-adjuvant, because there is no direct interaction between “adjuvant” and a vaccine.

The mechanism whereby laser pre-illumination augments immunity is completely novel and incompletely understood; one clear effect is the enhancement of the mobility of APCs, concomitant with little inflammation. An increase in the motility of these long branching dendrites of APCs promotes them to survey a greater area of the skin, thereby facilitating their antigen sampling as reflected by an increase in the number of AF670-OVA^+^DCs and MFI of OVA in individual cells at the site of injection. This unique behavior of DCs has been previously described as dendrite surveillance extension and retraction cycling habitude (dSEARCH) [25]. The increased motility may also contribute to the sufficient transportation of Ag-captured DCs to the draining LN. The mechanism of how laser illumination enhances the motility of APCs is not known at present but we offer the following rationale. In the skin, the initial lymphatic vessels are blind-end structures with wide lamina and thin walls. These initial lymphatic vessels drain excess fluid and solutes from the interstitial space and pass them to LN via lymphatic ducts. The draining process is extremely slow under normal physiological conditions but it can be increased as many as 10 times by inflammation or fever-range hyperthermia [26]; [27]. The interstitial space consists of a complex microarchitecture comprising fibrillar proteins and proteoglycans and offers the major barrier to molecular transport through the interstitium. Brief laser illumination may transiently alter the interstitial microarchitecture and increase the permeability and flow of interstitial macromolecules or cells to lymphatic capillaries as a result of photothermal and photomechanical effects. A fast interstitial flow may result in an increase in the number of Ag^+^DCs significantly not only in the 1° draining LN but also the 2° draining LN, due to increased lymphatic migration of Ag^+^DCs as well as a flow of free antigen from the skin to the draining LN via afferent lymphatic vessel. The latter may account primarily for the high number of Ag^+^DCs in the secondary draining LNs where free antigen from the skin was taken up by residue DCs.

A study reported in Russian that cutaneous laser exposure enhanced humoral immunity elicited by influenza vaccine delivered intradermally [28]. Those studies used a copper vapor laser that emitted a train of nanosecond pulses simultaneously at two wavelengths, 510 nm and 578 nm. Our laser emits a similar pulse train at the wavelength of 532 nm. Russian investigators used substantially higher laser power and density (0.6 W and 3 W/cm2) and longer exposure time (3 min) than we did (0.3 W, 0.78 W/cm2 and 2 min, respectively), with similar exposure spot sizes. The higher laser power and density stimulated persistent inflammation for about one month and production of extracellular HSP70 at the site of laser illumination [28]. In contrast, laser exposure in our study did not induce a significant inflammatory response as measured by the level of TNF-α, IL-6, IL-1β or CCL2 production. We also did not find an increase in either extracellular or intracellular level of HSP70 in the homogenate of laser-exposed skin over controls (data not shown). To the best of our knowledge, we report here the first non-inflammatory vaccine adjuvant, which can be potentially used to boost either Th1 or Th2 immune responses dependent of the nature of a given vaccine or the presence of other adjuvants.

To date, only two adjuvants (alum and MPL) have been approved for human use in the US, despite the fact that many potent vaccine adjuvants have been developed in animal studies. The major issue with the use of adjuvants for human vaccines is the concern on the potential toxicity and adverse side-effects of most of the adjuvant formulations since prophylactic vaccines are used to prevent illness, not treat diseases and much more stringent regulation is applied. LVA stands out as a unique technology and it is simple, convenient, risk-free, and cost-effective for vaccine dose-sparing and should thus warrant a further test in the clinic soon. The technology can potentially result in savings of a billion dollars a year in season flu vaccines alone and reduce the burden on the manufacturer of a new flu vaccine that is needed in sufficient quantity in a short time in an event of an outbreak of a new flu viral strain or pandemic.
